# Supplementary material for: Pseudouridine Synthase RsuA Confers a Survival Advantage to Bacteria under Streptomycin Stress
Source: Antibiotics (Basel). 2023 Sep 14;12(9):1447. doi: 10.3390/antibiotics12091447 (PMC10525438; doi:10.3390/antibiotics12091447)
Supplement: Supplementary file 1 [file antibiotics-12-01447-s001.zip › antibiotics-2537645-supplementary.pdf]

antibiotics

MDPI

Article

Pseudouridine Synthase RsuA Confers a Survival Advantage to Bacteria under Streptomycin Stress

Sudeshi M. Abedeera †, Kumudie S. Jayalath †, Jiale Xie, Rushdhi M. Rauff and Sanjaya C. Abeysirigunawardena *

Department of Chemistry and Biochemistry, Kent State University, 1175 Risman Dr., Kent, OH 44242, USA; asudeshi@kent.edu (S.M.A.); kjayalat@kent.edu (K.S.J.); jxie10@kent.edu (J.X.); mmohame1@kent.edu (R.M.R.)

* Correspondence: sabeysir@kent.edu

† These authors contributed equally to this work.

Supplemetary Information

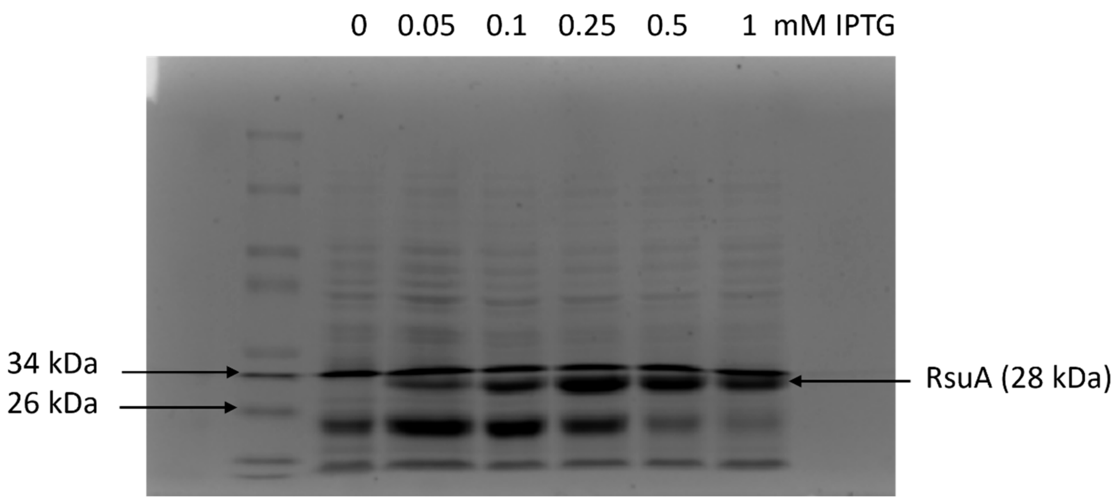

Supplementary Figure S1. RsuA expression level is visible on SDS PAGE gels at 0.05 mM inducer. RsuA overexpression under various IPTG concentrations are shown.

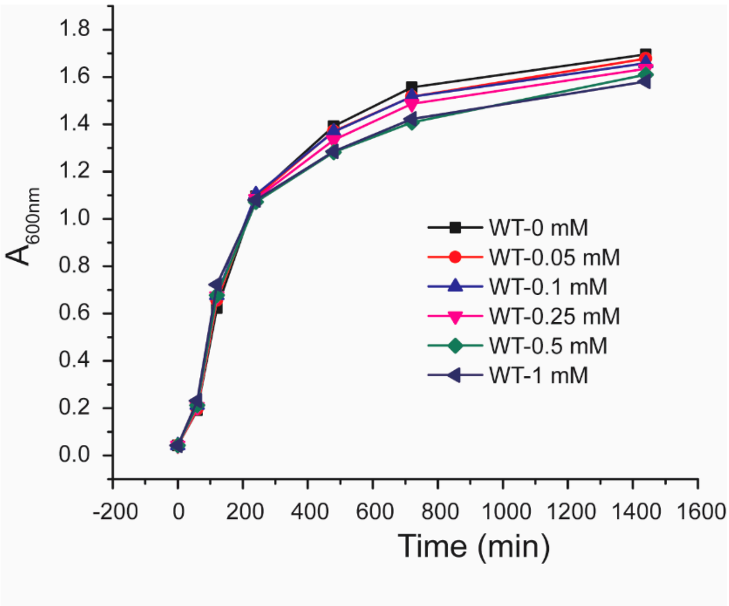

Supplementary Figure S2. The growth of E. coli is not significantly affected by the addition of IPTG. E. coli growth curves under various IPTG concentrations are shown.

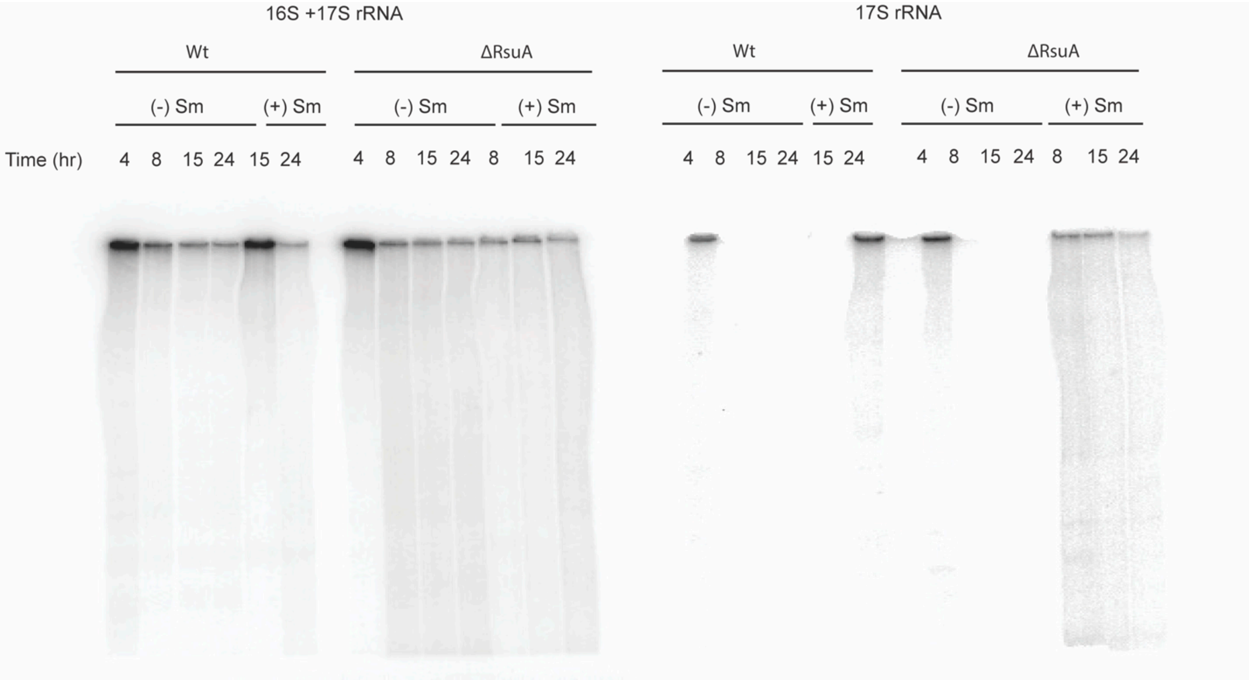

Supplementary Figure S3. Promer hybridization assay to determine 17S/16S rRNA ratio. Unique primers were annealed to 5′-leader of the 17S and mature 16S rRNA sequences. Native gel electrophoresis was performed for samples with rRNA-primer mixtures to separate the unbound primer. Primers were ^{32}P-labeled for visualization. All the experiments were performed in triplicates.

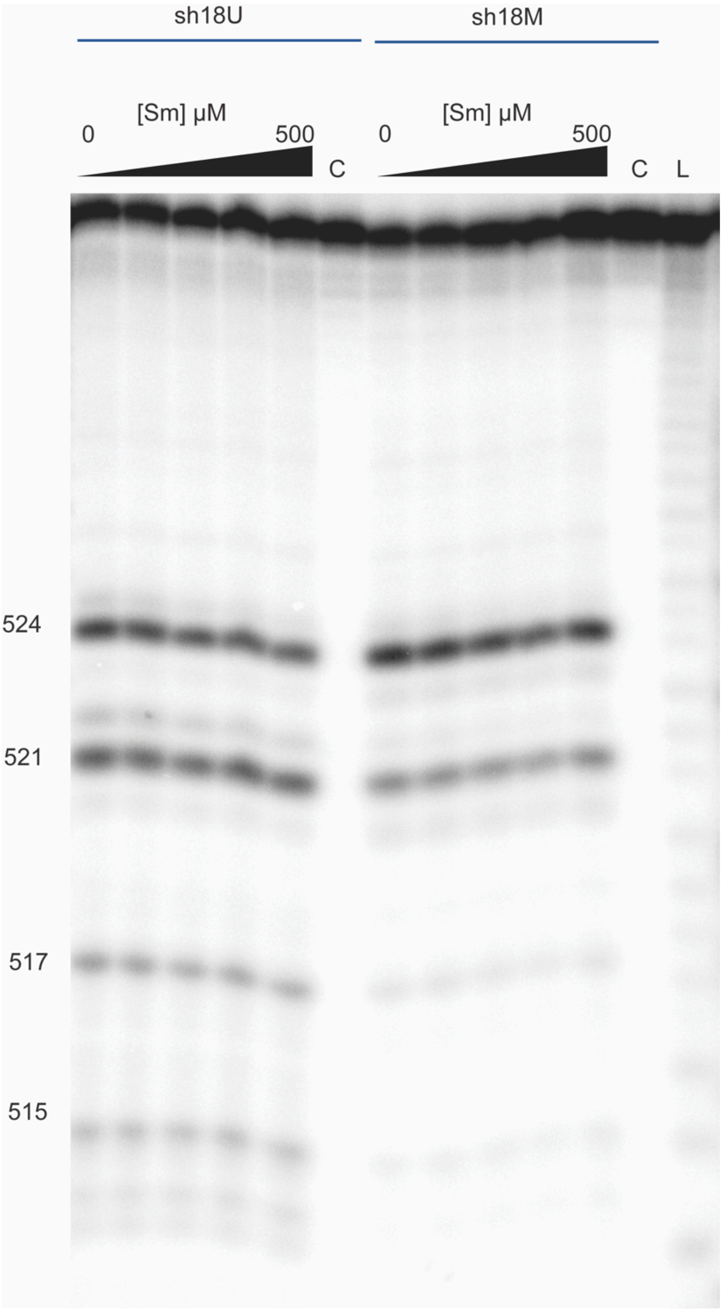

Supplementary Figure S4. RNase T1 footprinting of helix 18 model RNAs in the presence of streptomycin. Five streptomycin concentrations ranging from 0 μM – 500 μM were used. Lanes labeled as C and L represent control lanes with no RNase T1 digestion and alkaline hydrolysis ladder for helix 18, respectfully.

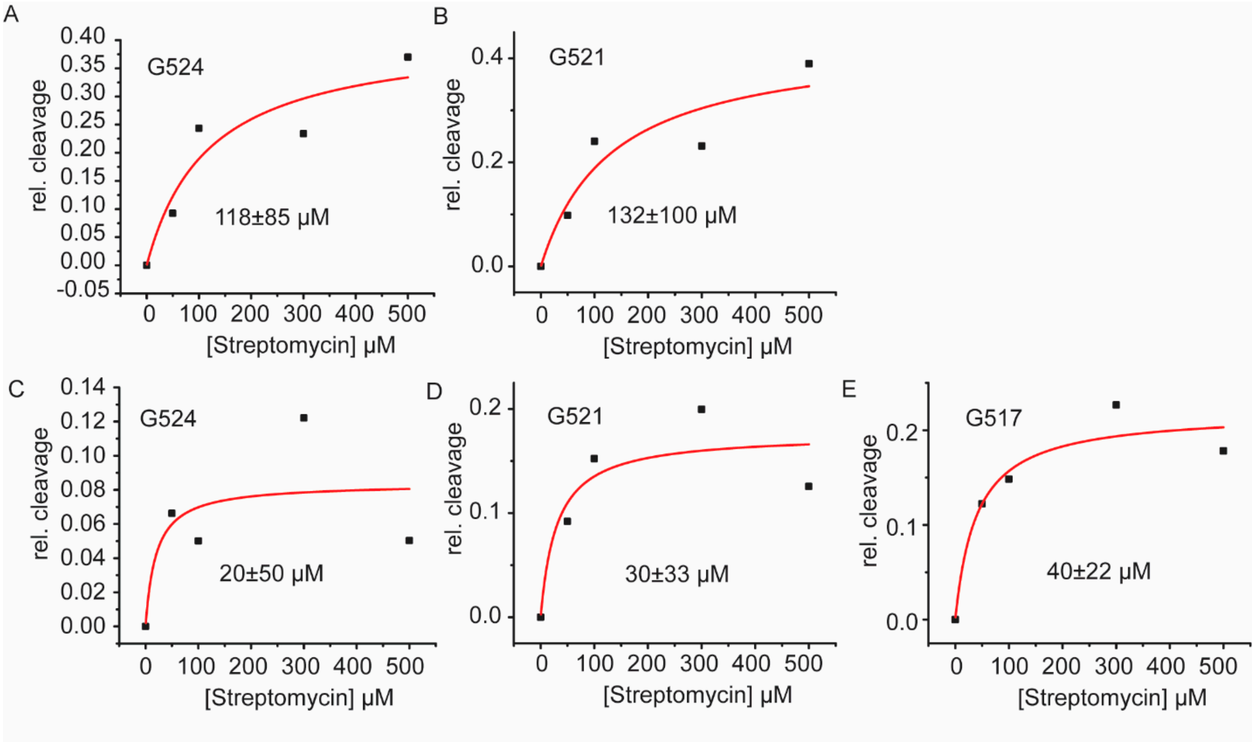

Supplementary Figure S5. Streptomycin binding to helix 18 model RNAs with different affinities. Binding curves were constructed using the RNase T1 cleavage for A, G524, B, G521 of modified helix 18 model RNA (h18-Ψ) and C, G524, D, G521, and E, G517 of unmodified helix 18 model RNA (h18-U). Binding curves are fitted to the binding isotherm equation to extract K_ds for Streptomycin-RNA interaction shown.

Supplementary Table S1. Growth rates of E. coli strains at various streptomycin concentrations.

[Strep] μg/mL	Wt		ΔRsuA	
	**1st phase**	**2nd phase**	**1st phase**	**2nd phase**
0	0.0109	-	0.0101	-
6	0.0089	-	0.0055	-
11	0.0043	0.0013	0.0037	-
13.5	0.0023	0.0018	0.0022	-
